# SARS-CoV-2 Vaccination as a Trigger for Perinuclear Antineutrophil Cytoplasmic Antibodies (p-ANCA) Associated With Rapidly Progressive Glomerulonephritis

**DOI:** 10.7759/cureus.29924

**Published:** 2022-10-04

**Authors:** Tábata Cano-Gámez, Javier Alejandro Teco-Cortes, María Virgilia Soto-Abraham, Everardo Álvarez-Hernández

**Affiliations:** 1 Rheumatology Department, Hospital General de México Dr. Eduardo Liceaga, Mexico City, MEX; 2 Nephropathology Department, Hospital General de México Dr. Eduardo Liceaga, Mexico City, MEX

**Keywords:** p-anca, vasculitis, rapidly progressive glomerulonephritis, vaccination, covid-19

## Abstract

The coronavirus disease 2019 (COVID-19) pandemic was devastating worldwide. The introduction of severe acute respiratory syndrome coronavirus 2 vaccination has reduced transmission, hospitalizations, and deaths, with infrequent major side effects. We present the case of a 51-year-old woman with rapidly progressive glomerulonephritis following COVID-19 vaccination with ChAdOx1 (AstraZeneca). Workup and histopathologic examination demonstrated active extracapillary proliferative lesions in cellular and fibrocellular crescents with extensive fibrinoid necrosis and karyorrhexis with diffuse glomerulonephritis, as well as positive perinuclear antineutrophil cytoplasmic antibodies. Treatment with cyclophosphamide and steroids was initiated with the improvement of renal function. Similar cases were seen with influenza vaccination, potentially describing vaccination as a possible trigger for anti-myeloperoxidase rapidly progressive glomerulonephritis.

## Introduction

The coronavirus disease 2019 (COVID-19) pandemic had a devastating impact on health and quality of life globally. To date, the World Health Organization has reported 514 million confirmed cases and more than 6 million deaths related to COVID-19 worldwide [1]. The introduction of severe acute respiratory syndrome coronavirus 2 (SARS-CoV-2) vaccination has reduced transmission, hospitalizations, and deaths, with infrequent major side effects [2]. Proving a causal relationship between side effects and vaccination is challenging, and few studies have reported kidney diseases after SARS-CoV-2 vaccination, with heterogeneous histopathologic findings [3].

Antineutrophil cytoplasmic antibody (ANCA)-associated vasculitis is a rare group of autoimmune diseases causing inflammatory cell infiltration and necrosis of blood vessels. Although clinical presentation may vary, patients frequently present with kidney involvement requiring prompt diagnosis and treatment [4]. During the influenza vaccination campaign, several reports associated vaccination with ANCA vasculitis, stating its possible role as a trigger for disease presentation [5].

We report the case of a patient who developed perinuclear antineutrophil cytoplasmic antibodies (p-ANCA) associated with rapidly progressive glomerulonephritis after ChAdOx1 (AstraZeneca) SARS-CoV-2 vaccination.

## Case presentation

A 51-year-old woman with a medical history of secondary hypothyroidism was evaluated for fever, nausea, asthenia, hyporexia, myalgia, and polyarthralgia, predominantly in the knees following her third dose of ChAdOx1 (AstraZeneca) SARS-CoV-2 vaccine. She had no relevant kidney disease history, and her laboratory results before vaccination were unremarkable.

She was managed by a general practitioner with symptomatic treatment, although her symptoms persisted and she reported a weight loss of 7 kg over the course of a month. Laboratory findings demonstrated creatinine levels of 1.3 mg/dL, urea of 39 mg/dL, and urinalysis with the presence of abundant leukocytes and mild proteinuria. Treatment with ceftriaxone and paracetamol was initiated, without further improvement. One week later, laboratory findings showed creatinine levels of 3.99 mg/dL and urea of 99 mg/dL, for which she was referred to our emergency department. After 24 hours, creatinine increased to 4.98 mg/dL and urea to 114 mg/dL.

The patient’s clinical examination was unremarkable, without any particular skin finding or edema. Complementary studies reported negative antinuclear antibodies, negative anti-double-stranded DNA (anti-dsDNA) antibodies, normal complement levels, negative c-ANCA and anti-myeloperoxidase (MPO) antibodies with high titers (50.89 U/mL, positive >15 U/mL). Urinalysis reported proteinuria of 743 mg in 24 hours and active urinary sediment with 20-40 leukocytes/high-power field (HPF), hyaline casts (2-5 per/HPF), and >41 erythrocytes/HPF with 36% dysmorphia. P-ANCA-associated rapidly progressive glomerulonephritis was considered and treatment with methylprednisolone pulses was initiated. During follow-up, the patient developed uremic syndrome, and renal replacement therapy was started (Table 1).

**Table 1:** Patient’s laboratory findings before and after vaccination.

Days post-vaccination	-20	+10	+26	+30	+31	+32	+33	+34
Serum creatinine (mg/dL)	0.53	1.3	3.99	4.98	4.87	4.64	4.62	2.9
Serum urea (mg/dL)	11	15.5	99	114	136	148	154	78
Intervention				Methylprednisolone			Cyclophosphamide	Hemodialysis

**Figure 1:**
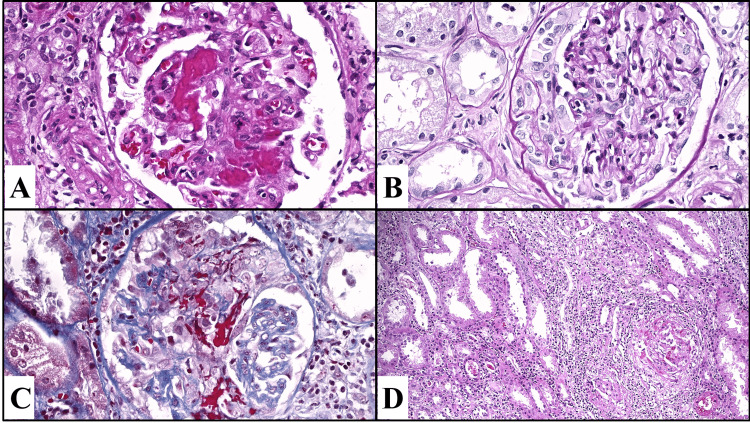
(A) Glomeruli with extensive segmental fibrinoid necrosis along with karyorrhexis. (B) Active extracapillary proliferative lesions (cellular and fibrocellular crescents). (C) Some glomeruli show both fibrinoid necrosis and active extracapillary proliferative lesions, with segments of the capillary tufts. (D) Extensive interstitial inflammatory infiltrate composed of lymphocytes, plasma cells, neutrophils, and eosinophils.

Renal biopsy reported 87% of the glomeruli with active extracapillary proliferative lesions in cellular and fibrocellular crescents with extensive fibrinoid necrosis and karyorrhexis, as well as glomeruli with rupture of Bowman’s capsule and extension of the inflammatory process to the interstitium (Figure 1). Diffuse extracapillary proliferative glomerulonephritis was concluded, with segmental fibrinoid necrosis, active tubulointerstitial nephritis, multifocal acute tubular injury with moderate regenerative changes of the tubular epithelium, and grade I interstitial fibrosis.

Cyclophosphamide 1 g, prednisone 1 mg/kg, and mycophenolate mofetil 2 g were started as induction therapy soon after methylprednisolone pulses, following the British Society protocol for systemic vasculitis management. After treatment, the patient’s clinical status improved and creatinine lowered to 2.9 mg/dL. During outpatient follow-up, the renal function continued to improve in the next three months with no further hemodialysis requirements and with mycophenolate mofetil as maintenance therapy.

## Discussion

Emergency approval of SARS-CoV-2 vaccination was necessary to reduce mortality and contagion due to the COVID-19 pandemic. Surveillance programs were established globally to notice any major adverse effects related to vaccination. As cases occurred within close temporality, assumptions may be made regarding association [6]. There have been many studies and epidemiological reports regarding adverse events following SARS-CoV-2 immunization. A community-based study in the United Kingdom investigated the adverse effects following the administration of the ChAdOx1 vaccine in 345,280 individuals. The study found systemic side effects in 33.7% and local side effects in 58.7% after the first dose of ChAdOx1 nCoV-19. However, the study did not report major adverse effects [7]. On the other hand, case reports of major adverse effects with renal involvement have been published since the beginning of SARS-CoV-2 immunization in 2020. Muhammad et al. described the case of a 78-year-old woman who developed new-onset renal-limited ANCA-associated vasculitis following Pfizer-BioNTech vaccination [8]. Further, a case of a 79-year-old woman with massive rhabdomyolysis and pauci-immune crescentic glomerulonephritis after Pfizer-BioNTech COVID-19 mRNA vaccination was reported by Hakrous et al. in 2021 [9]. Autoimmune adverse effects have been described in a few cases. Although autoimmune events have been observed after vaccination, we cannot establish a cause-effect. Proposed mechanisms include molecular mimicry, epitope spreading, and increased autoreactive T cell production.

Nevertheless, the association between RNA vaccines and the development of autoantibodies and autoimmune rheumatic diseases such as ANCA-associated pathologies is not new and has already been reported during the influenza A-H1N1 pandemic. In 2015, Jeffs et al. presented the case of a previously healthy patient who developed ANCA-associated vasculitis shortly after influenza vaccination. Based on this, they concluded that the presence of viral RNA in influenza vaccines was capable of activating the immune system, providing evidence for a possible role as a trigger for autoimmune diseases [10]. The temporal association to RNA or viral vector vaccines suggests the vaccine as a trigger in predisposed individuals as is the case for some neurological adverse effects such as optic neuritis and transverse myelitis [11,12].

Although the direct association between vaccination and the development of rapidly progressive glomerulonephritis cannot be demonstrated, it is possible to establish a temporal relationship and possibly identify the vaccine as a trigger for the development of autoimmune pathologies.

## Conclusions

The COVID-19 vaccine cannot be demonstrated as a direct cause of kidney disease; however, due to the temporal association between the application of ChAdOx1 nCoV-19 vaccine and the onset of renal function decline in a previously healthy patient, it is possible to establish a causal relationship. In addition, there have been reports of similar cases after the administration of RNA vaccines, which reinforces the association. Surveillance of vaccination-associated reactions should continue and this factor should be considered in patients with an epidemiological history of vaccination who present with an acute decline in renal function.
